# Mesenchymal stem cells and exosomes in ischemic brain injury: a review

**DOI:** 10.3389/fgene.2025.1639756

**Published:** 2025-08-29

**Authors:** Haiyan Xu, Lanlan Yang, Weitie Wang, Chengwei Zhang

**Affiliations:** ^1^ Department of Rehabilitation Therapeutics, School of Nursing, Jilin University, Changchun, Jilin, China; ^2^ Department of Biliary and Pancreatic Internal Medicine, The Second Hospital of Jilin University, Changchun, Jilin, China; ^3^ Department of Cardiovascular, The Second Hospital of Jilin university, Changchun, Jilin, China; ^4^ Department of Anesthesiology, The Second Hospital of Jilin university, Changchun, Jilin, China

**Keywords:** mRNA, exosome, review, MSc, Treatment

## Abstract

Stroke poses a serious threat to human health and life, serving as a leading cause of death and disability in adults. The incidence rate of stroke continues to rise annually. Following the onset of ischemic stroke, most patients experience a period of spontaneous recovery. Neural repair after cerebral ischemia is closely associated with neurovascular plasticity, which facilitates the regeneration and repair of nerves and blood vessels in the ischemic injury area. Mesenchymal stem cells (MSCs), adult stem cells isolated from bone marrow or other tissues, can differentiate into various cell types and possess characteristics such as self-renewal, low immunogenicity, and easy of isolation. Exosomes are regarded as the primary mediators of MSC functions. These specialized extracellular vesicles play critical roles in intercellular communication, targeted transport, and regulation of recipient cell functions through their surface molecules and cargo (e.g., proteins, RNA, and other bioactive factors). Studies demonstrate that MSCs and their exosomes participate in both neuronal and vascular endothelial cell damage and repair after stroke. They exert distinct effects at different stages of cerebral ischemia injury, promoting angiogenesis, neurogenesis, and reducing inflammation. While preclinical studies show promising therapeutic potential, clinical translation faces challenges such as standardization of exosome isolation, optimal dosing, delivery methods, and long-term safety evaluation. Future research should focus on overcoming these barriers to facilitate their application in stroke therapy. This review summarizes current research on the therapeutic potential of MSCs and their exosomes in ischemic brain injury.

## 1 Introduction

In recent years, stroke has emerged as the leading cause of mortality and disability worldwide. Ischemic stroke accounts for 80%–90% of stroke cases (Paul and Candelario-Jalil, 2021). Cerebral ischemia-reperfusion injury occurs when cerebral tissue suffers damage due to ischemia, followed by further exacerbation upon blood flow restoration and reperfusion. The underlying mechanisms may involve oxygen free radical activation of lipid peroxidation, protein denaturation, mitochondrial apoptosis, and death receptor signaling during reperfusion. Mesenchymal stem cells (MSCs) are multipotent cells capable of self-renewal and under specific conditions, differentiation into various functional cells. These cells exhibit repair, anti-aging, and anti-inflammatory properties. Research indicates that MSCs primarily exert their biological functions through exosomes. Exosomes are specialized extracellular vesicles secreted by most cells (Liu et al., 2021). They have a diameter of 40–100 nm and utilize their surface-specific molecules such as heat shock protein (HSP), Alix, white blood cell differentiation antigen 68 (CD68), as well as internal proteins, microRNAs, long chain non-coding RNAs, and DNA to facilitate intercellular information exchange and regulate target cell function (Lai et al., 2022). Recent studies reveal that exosomes secreted under different stress conditions exhibit distinct characteristics and functions, making them potential dynamic biomarkers for stroke progression (Lee et al., 2022). Additionally, exosomes serve as efficient natural carriers for targeted drug delivery and participate in cerebrovascular repair. The microRNAs within exosomes further modulate recipient cell functions (Xu et al., 2022; Javadi et al., 2022). Currently, exosomes play critical roles as damage markers, therapeutic carriers, and regulators of cellular repair in cardiovascular and cerebrovascular diseases. Notably, studies have found that RNAs carried by exosomes could serve as novel biomarkers for different stages of brain ischemia. This article reviews the roles of stem cells and exosomes in the pathogenesis, progression, and treatment of ischemic stroke.

## 2 Neurorepair after cerebral ischemia

During cerebral ischemia, both nerves and blood vessels react simultaneously, making treatments focused solely on neuroprotection inefficient (Table 1). It is necessary to consider nerves and blood vessels as an integrated system. The neurovascular unit (NVU) is a complex cellular system composed of neurons, interneurons, astrocytes, smooth muscle cells covered by the basal layer, pericytes, endothelial cells, and extracellular matrix (ECM), where interactions between these components maintain brain microenvironment stability and function (Wang Q. et al., 2023). Endothelial cells anchored to the basement membrane, and surrounded by astrocyte end-feet connect via tight junction proteins to form the blood-brain barrier (BBB), which serves as the NVU’s core interface between cells and matrix. Under both physiological and ischemic conditions, the BBB is dynamically regulated by NVU components to optimize cerebral blood flow (Yang Q. et al., 2022). Cerebral ischemia triggers self-repair processes, primarily angiogenesis and neurogenesis. Angiogenesis after cerebral ischemia refers to the production of new blood vessels from existing endothelial cells to restore nutrients and oxygen supply. After cerebral ischemia, the microvascular density in the penumbra significantly increases with the prolongation of ischemia time (Kanazawa et al., 2019). This neovascularization enhances blood supply to ischemic tissue, restores the cellular metabolism of surviving neurons, promotes neural repair after ischemia, and provides necessary nutritional support for newborn neurons. The increase in neovascularization is usually closely related to the reduction of infarct volume and improvement of neural function caused by ischemia, which is an important indicator for measuring nerve repair. The ischemic site first undergoes angiogenesis, followed by axonal growth, and the generation of new blood vessels to promote neurogenesis (Kanazawa et al., 2017). Neurogenesis is a process by which endogenous neural stem cells (NSCs) and neural progenitor cells (NPCs) generating new functional neurons, including proliferation, migration and differentiation into mature neurons. Throughout life, neurogenesis continues in two different regions: the subventricular zone (SVZ) of the lateral ventricle and the subgranular zone (SGZ) of the dentate gyrus of the hippocampus. In stroke animal models and patients, studies have found an increase in neurogenesis after cerebral ischemia injury (Lindvall and Kokaia, 2015). Ischemia injury upregulates cytokines, such as fibroblast growth factor-2, insulin-like growth factor-1 (IGF-1), brain-derived neurotrophin factor (BDNF) and VEGF, promoting proliferation and migration from SVZ to the ischemic striatum. Angiogenesis after cerebral ischemia can also enhance the neural repair process. The new microvessels in the penumbra can provide oxygen, nutrients and cytokines. Endothelial cells in the striatum secrete BDNF to create a permissive microenvironment for NSC migration, while microvessels express β1 integrin to guide NSC adhesion and migration along vascular scaffolds (Fujioka et al., 2017). After reaching injured areas, NSCs differentiate into neurons, followed by axonal sprouting and growth, ultimately promoting nerve repair (Fujioka et al., 2017).

**TABLE 1:** Neurorepair process after cerebral ischemia.

Phase	Core events	Key molecules
Acute Phase (Hours-Days)		
Ischemic Injury	Blood flow disruption → Hypoxia, ATP depletion → Neuronal cell death	Glutamate excitotoxicity ROS (Jiang et al., 2020)
Early Repair Signals	Activation of astrocytes/microglia→ Release of cytokines and growth factors	TGF-β, IL-10, BDNF, NGF, VEGF (Mayfield et al., 2016)
Subacute Phase (Days-Weeks)		
Clearing Debris	Microglia/phagocytes remove dead cells→Astrocytes form a glial scar	GFAP, S100β, CX3CR1 Iba1
Angiogenesis	VEGF-driven formation of new blood vessels to restore perfusion	VEGF-A, PDGF, Angiopoietin-1
Neurogenesis	Neural stem cells in the subventricular zone→ Hippocampus migrate to damaged areas	BDNF, IGF-1, DCX, MMP-9
Chronic Phase (Weeks-Months)		
Axonal Sprouting	Surviving neurons extend axons/dendrites→ Synapse reorganization	Netrin-1, BDNF, Nogo-A, GAP-43, STAT3
Remyelination	Oligodendrocyte precursor cells differentiate to repair damaged myelin	CaMKII, CREB, PSD-95, Synaptophysin
Functional Recovery	Cortical remapping	Olig2, Myelin Basic Protein

## 3 MSCs promote neural repair after cerebral ischemia

### 3.1 Overview of MSCs

MSCs are stem cells with multilineage differentiation potential that can be isolated from bone marrow and other tissues according to the International Society for Cellular Therapy criteria. They can proliferate in other tissues and have the following three characteristics (Sheng, 2015): ① plastic-adherent under standard culture. ② Expression of CD73, CD90, and CD105, but lacking CD45, CD34, CD14, or CD11b, CD79 α, CD19 and the surface molecules of human leukocyte antigen-DRisotype. ③ Ability to differentiate into osteoblasts, adipocytes, and chondrocytes *in vitro*. MSCs reside in the stroma of many adult tissues. They can be found and isolated from various tissues, such as bone marrow, adipose tissue, umbilical cord blood, brain, kidney, lungs, and muscle, with differentiation potential into osteoblasts, adipocytes, chondrocytes, muscle cells, and neurons. MSCs represent approximately 0.001%–0.01% of bone marrow cells, making bone marrow the most important source for MSC isolation (Jin and Lee, 2018). Substantial evidence indicates that MSCs promote functional recovery after cerebral ischemia through multiple distinct mechanisms (Pittenger et al., 2019; Jaillard et al., 2020).

### 3.2 MSCs promote angiogenesis after cerebral ischemia

Microvascular angiogenesis enhances cerebral blood flow and nutrient delivery to the ischemic regions. During this process, the initial vascular plexus forms mature blood vessels through germination, branching, pruning, endothelial cell proliferation, and perivascular cell recruitment, with post-ischemic angiogenesis being formed from the original blood vessels branches. Both angiogenesis and vascular maturation are coordinately regulated by VEGF signaling and its receptors, as well as the angiopoietin 1 (Ang1)/Tie2 system (Pang et al., 2018). Experimental studies demonstrate that after using the middle cerebral artery occlusion (MCAO) model to induce cerebral ischemia in rats, MSCs were immediately injected into the brain through the internal carotid artery. After 7 days, the expression of VEGF in the brain is significantly increased and promoted vascular remodeling (Lapi et al., 2015). Similarly, in human MSCs transplantation within 24 h post-ischemia in rat models, the expression of VEGF, Ang1 and Tie2 in the ischemic region increased, and the stability of neovascularization improved post-stroke neural repair (Moisan et al., 2016). Research on MSC-mediated angiogenic therapy reveals synergistic effects when combined with pharmacological agents. Co-administration of MSCs with icaritin reduces the volume of cerebral infarction in rats with cerebral ischemia through phosphatidylinositol 3-kinase (PI3K) and extracellular signal-regulated protein kinase 1/2 (ERK1/2) pathways. These pharmacological agents significantly upregulate the expression of VEGF and BDNF, and synergistically promoting angiogenesis and neurogenesis after cerebral ischemia (Liu et al., 2018). Furthermore, combined mannitol and temozolomide combination therapy effectively delivers the microcapsules released by human umbilical cord MSCs to the brain of MCAO model rats, and promotes angiogenesis at the injured site (C et al., 2018). These findings establish that pharmacological augmentation of MSCs therapy not only enhances therapeutic efficacy but also represents a promising direction for advancing MSC-based stroke treatments.

### 3.3 MSCs promote neural repair after cerebral ischemia

The plasticity of neurons following cerebral ischemia reflects the brain’s intrinsic repair capacity and is also related to the increasing number of other cells in the brain (such as astrocytes and microglia) (de Oliveira et al., 2023). MSCs enhance brain plasticity after cerebral ischemia by releasing nutrients and growth factors such as VEGF, BDNF, and bFGF, while also promoting neural repair by inhibiting the expression of pro-inflammatory factors. These MSC-derived factors not only promote the generation of blood vessels, but also affect the process of neural repair, including improving neuronal plasticity, promoting the recovery of glial cell function, and reducing brain inflammation, thus playing a role in the NVU as a whole (Pittenger et al., 2019).

#### 3.3.1 MSCs promote neural progenitor cell migration and neurogenesis

In both the SVZ of ischemic stroke patients and focal cerebral ischemia animal models, the brain’s endogenous regenerative ability of the brain is not sufficient to repair brain injury despite NPCs proliferation. Therefore, promoting the proliferation and migration of endogenous NSCs and NPCs to the injured area, as well as transplanting NSCs to assist endogenous NPCs, are effective strategies for repairing cerebral ischemic injury (Du et al., 2024). BDNF is a member of the neurotrophic protein family, plays a pivotal role in the regulation of neuroprotection, neurogenesis and neuroplasticity, and serves as a key medium for rehabilitation after ischemic stroke. After cerebral ischemia, the expression of BDNF and VEGF in brain tissue increases, promoting endogenous NSC proliferation. Experimental studies demonstrate that MSC transplantation into ipsilateral brain tissue of MCAO model rats elevates the expression of BDNF, recombinant human neurotrophic protein-3, and VEGF in ischemic brain tissue, significantly enhancing NSC proliferation (Hamada et al., 2005). Stromal cell-derived factor-1α (SDF-1 α), chemokine receptor 4 (CXCR4) have been shown to guide the migration of stem cells to the injury area. The polysialylated nerve cell adhesion molecule (PSA-NCAM) expressed in NPC can promote the migration of NPC to the injured area. MSC transplantation in cerebral ischemia rats upregulates SDF-1 expression (Shiota et al., 2018), consequently increasing the number of NPC with positive expression of PSA-NCAM-positive NPCs in the ischemic brain area-confirming MSCs’ dual role in promoting both NPC proliferation and migration post-ischemia.

#### 3.3.2 MSCs reduce neuroinflammation

Multiple studies indicate that the level of matrix metalloproteinase 9 (MMP-9) is significantly increased in the ischemic brain of mice, rats and ischemic stroke patients, which is mainly secreted by invasive neutrophils, and induced BBB damage (Rosenberg et al., 2001). MSC-based interventions show notable neuroprotective effects, when administered intracerebrally to ischemic mice, MSCs reduce BBB damage while decreasing interleukin-1β (IL-1β) and other pro-inflammatory cytokine levels. A study also found that MSCs can inhibit the inflammatory reaction and reduce the damage to the BBB by inhibiting the expression of cell adhesion factor-1 in endothelial cells, reducing neutrophil infiltration, and down regulating the expression of MMP-9 (Cheng et al., 2018). The detrimental impact of chronic post-ischemic inflammation on neural repair has been further demonstrated in MCAO model rats receiving intravenous MSC injections at 60 days post-ischemia. The results showed that MSCs can significantly reduce the number of activated microglia in the brain and improve the neuroinflammatory response induced by cerebral ischemia (Cui et al., 2022).

## 4 MSCs promote nerve repair through exosomes

**FIGURE 1:**
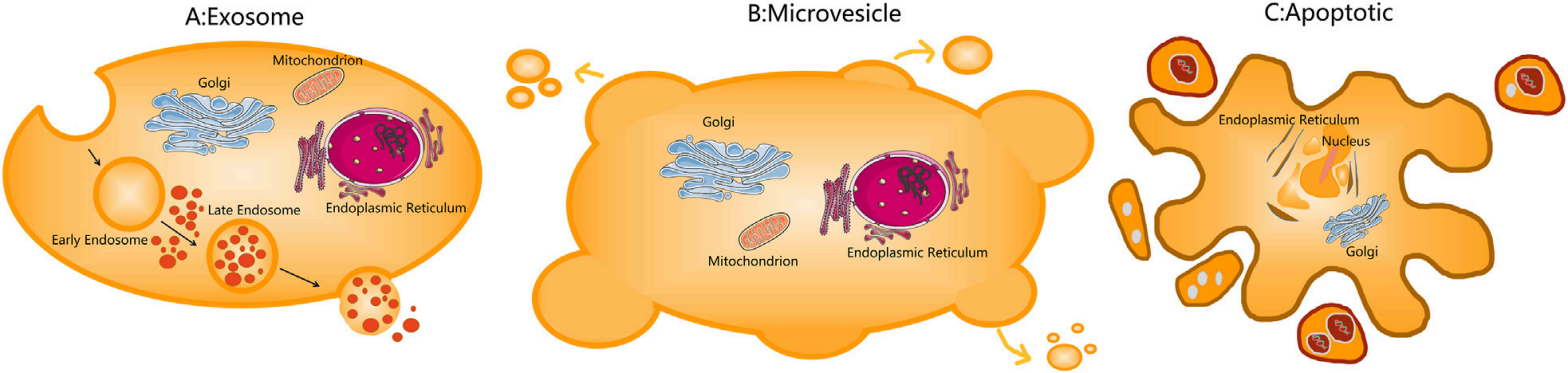
Schematic of extracellular vesicles. **(A)** Exosomes are extracellular vesicles of endocytic origin derived from different cells. **(B)** Microvesicles are derived from the plasma membranes of cells. **(C)** Apoptotic bodies are vesicles that separate from post-apoptotic cells.

**FIGURE 2:**
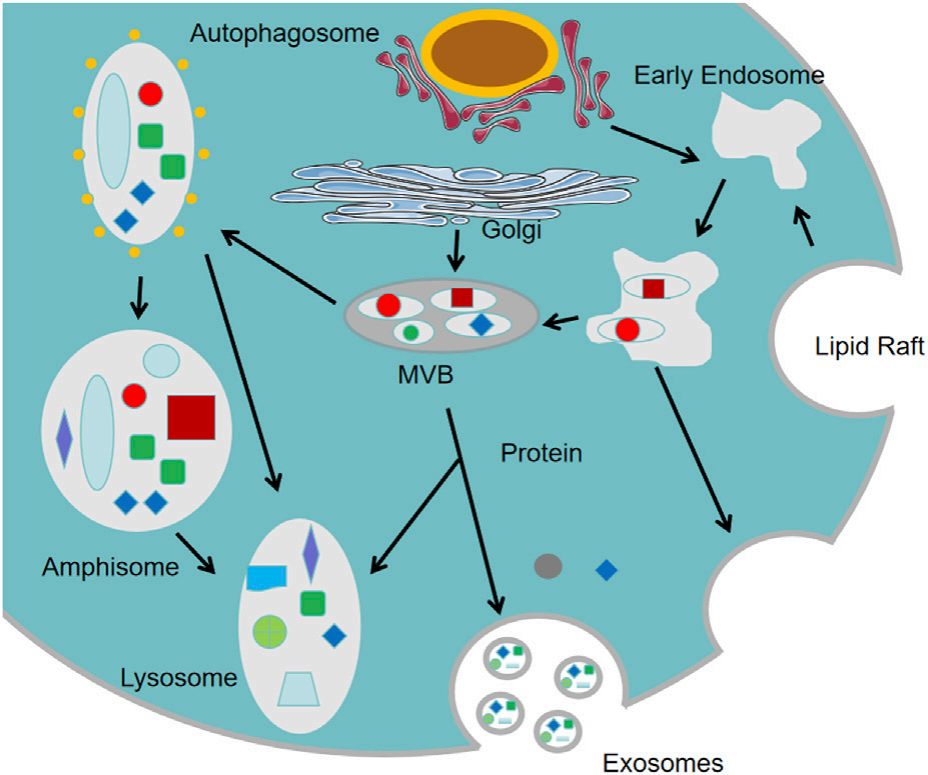
Biogenesis of exosomes. Exosomes originate from membrane invagination, which leads to the formation of multivesicular bodies and fusion with the plasma membrane, resulting in the release of exosomes.

In ischemic stroke research, intravenously injected MSC-derived vesicles exhibit significant neuroprotective effects, demonstrating that MSC-mediated neurological recovery is not solely dependent on cell replacement but also involves potent paracrine mechanisms. Recent years have seen a significant increase in research on extracellular vesicles (EVs), which are classified into exosomes, microvesicles, and apoptotic bodies based on size (Figure 1). Xin et al. (2017a) found that MSCs overexpressing miR-133b enhance neural repair in MCAO-induced ischemic rats. The exosomes derived from MSCs overexpressing miR-133b further enhance neural repair in MCAO-induced ischemic rats. Research showed that the exosomes secreted by MSCs transfected with miR-17–92 had a stronger ability to promote neural function recovery compared to those secreted by untransfected MSCs (Xin et al., 2017b). These studies suggest that exosomes and the microRNAs originating from exosomes may be key to promoting neural repair during MSCs treatment. Exosomes are nanoscale extracellular vesicles secreted via exocytosis from cells (Figure 2). The direct use of exosomes for the treatment of cerebral ischemia and nerve injury may be more beneficial than the use of MSCs alone. The application of exosomes can overcome many disadvantages associated with MSCs therapy, such as abnormal differentiation of MSCs and the formation of teratomas (Aguiar Koga et al., 2023).

Exosomes possess the ability to cross the BBB, making them highly valuable as drug delivery vehicles for neural repair applications. The BBB, primarily located in brain microvessels, serves as the brain’s protective barrier by maintaining cerebral homeostasis and blocking exogenous threats and circulating harmful substances. Exosomes, characterized by their bilayer lipid membrane structure, can traverse the BBB bidirectionally. Five primary mechanisms have been proposed for this transport (Sa et al., 2020): (1) interaction with G-protein coupled receptors; (2) membrane adhesion and fusion; (3) macropinocytosis; (4) nonspecific diffusion via lipid rafts; or (5) receptor-mediated transcytosis.

### 4.1 The role of exosome in cerebral ischemic injury

As a medium for intercellular signal exchange, exosomes have a close relationship with cerebral ischemic injury (Table 2). Research has demonstrated that serum exosomal miR-328-3p levels are markedly elevated in stroke patients with poor short-term prognosis compared to those with favorable outcomes (Wang et al., 2021). Cerebral ischemic injury model rats treated with serum exosomes from patients with poor prognosis showed exacerbated cerebral ischemic injury. Therefore the miR-328-3p in the exosomes may serve as one of the biological indicators for short-term assessment of the degree of brain injury (Wang et al., 2021). Researchers have isolated exosomes from serum of rats subjected to MCAO-reperfusion and performed proteomic analysis. The results revealed that growth arrest and DNA damage-inducible protein 34 (GADD34) in the exosomes significantly increased. Administration of GADD34 inhibitors could alleviate cerebral ischemic injury in rats (Yang et al., 2021). Ye et al. (Ye et al., 2020) isolated and identified exosomes from acute cerebral infarction and normal human serum. Injecting exosomes from acute cerebral infarction patients into MCAO/R model rats resulted in increased brain injury, poor behavioral recovery, and promoted neuroinflammation.

**TABLE 2:** Function of exosomes in ischaemic stroke.

Original	Model	Time	Function
Neural stem cell	Thromboembolic-MCAO	2 h	Immunomodulation, inhibit inflammation (Jiang et al., 2020)
	1 h MCAO	2 h	Preserve astrocyte function (Mayfield et al., 2016)
	Permanent MCAO	2 h	Protect the integrity of BBB (Jiang et al., 2020)
Adipose-derived stem cell	50 min MCAO	3 h	Anti-inflammation, antiapoptosis (Li et al., 2018; Gao et al., 2023)
	60 min MCAO	3 days before	Promote autophagy (Li et al., 2018; Gao et al., 2023)

MCAO: middle cerebral artery occlusion; BBB: blood-brain barrier.

At the cellular level, the glucose oxygen deprivation/reperfusion (OGD/R) model induces dysregulated miRNA expression in exosomes secreted by rat cortical neurons. These neuron-derived exosomes exhibit concentration-dependent neurotoxic effects, reducing neuronal viability, promoting apoptosis, and significantly impairing dendrite formation (Hsu et al., 2020). OGD/R pretreatment of rat small intestinal crypt epithelial cells co-cultured with cortical neuronal cells can lead to decreased synaptic toxicity, and activity, and neuronal apoptosis of cortical neurons (Hsu et al., 2020). These findings indicate that during brain injury, cortical neuron-derived exosomes may encapsulate pathogenic factors which, upon internalization by recipient cells, exacerbate neuronal damage via pro-apoptotic and synaptotoxic pathways (Wang et al., 2021; Yang et al., 2021; Ye et al., 2020; Hsu et al., 2020). Consequently, exosomes can effectively improve the symptoms of cerebral ischemic injury.

The local inflammatory response exacerbates brain damage through increased vascular permeability, which facilitates leukocyte migration and exudation. In recent years, the repair and regeneration of cerebral blood vessels following cerebral ischemia have emerged as a research hotspot. Both *in vitro* and *in vivo* studies confirm that endothelial progenitor cells can secrete miRNAs associated with the phosphatidylinositol 3-kinase/protein kinase B (PI3K/Akt) signaling pathway. Pro-angiogenic miRNAs, including miR-126 and miR-296, play a critical role in vascular repair by promoting endothelial cell-mediated regeneration through PI3K/Akt pathway activation. (Dai et al., 2018; Chen et al., 2015). Xu et al. (Xu et al., 2017) demonstrated that neurons acted on the Eef2k gene in vascular endothelial cells by secreting exosomes containing miR-132. By inhibiting the expression of the Eef2k gene, they upregulated the expression of Cdh5 molecules and enhanced vascular integrity. Glioblastoma-derived exosomes similarly influence vascular endothelial cells by delivering pro-angiogenic proteins, mRNAs, and miRNAs, a mechanism that appears conserved in cerebral ischemia. Furthermore, stressed vascular endothelial cells release exosomes containing VEGF-B that are internalized by damaged endothelial cells. This process amplifies VEGF expression in recipient cells, promoting vascular repair through autocrine and paracrine mechanisms (Couch et al., 2017).

Studies of myocardial ischemia-reperfusion injury have revealed that miR-223-3p in the exosomes regulates the ribosome protein S6 kinase Β 1/Oxygen inducible factor 1 α signaling pathway to inhibit the repair of cardiac microvascular endothelial cells (Mayfield et al., 2016). Whether this mechanism also exists during cerebral ischemia and the specific mechanism are worth further research (Suzu et al., 2017). Following stroke, there is a certain correlation between the size of the ischemic penumbra and the integrity of cerebral blood vessels. Reducing the penumbral area and promoting vascular repair are crucial for improving stroke outcomes and preventing recurrence. Emerging evidence demonstrates that exosomal miRNAs derived from both neurons and vascular endothelial cells play pivotal roles in regulating vascular structural integrity and facilitating angiogenic repair processes. The mechanisms underlying exosome-mediated vascular repair and regeneration following cerebral ischemia involve complex intercellular communication. Subependymal neural stem cells dynamically interact with both peripheral neurons and vascular endothelial cells through continuous activation of multiple signaling pathways, demonstrating pluripotent differentiation potential. Therefore, more and more research focuses on study the signaling pathways (ERK/MAPK, mTOR, β-Catenin) in different differentiation directions of subependymal neural stem cells (Iroegbu et al., 2021). Current evidence indicates that exosomes derived from glial cells and exosomes in cerebrospinal fluid participate in regulating the function and immune response of neural cells by regulating signaling pathways within neural stem cells (da Cruz et al., 2020). Through analysis of rat and human cerebrospinal fluid exosomes, these exosomes contain key proteins and related miRNAs in the insulin-like growth factor signaling pathway. These exosomes actively regulate neural stem cells differentiation into neuronal cells, revealing a potential therapeutic mechanism for clinical diagnosis and treatment of cerebral ischemia. This discovery will provide new ideas for promoting neurogenesis to replace neurons lost to ischemic injury (Feliciano et al., 2014). Another study found that using pro-inflammatory cytokines to stimulate neural stem cells *in vitro* could promote the secretion of exosomes rich in miRNA by neural stem cells. By acting on target genes of vascular endothelial cells, it upregulated the expression of γ-interferon and further activated signal transducer and transcriptional activator signaling pathways, reducing the inflammatory response, and improving the activity of damaged neuronal cells (Noori et al., 2021). Overexpression of ionic glutamate receptors (NMDA) during cerebral ischemia could exacerbate neuronal damage (Liu et al., 2023). Studies demonstrate that exosomal miR-233 controls the response to neuronal injury by regulating the functional expression of the glutamate receptor subunits GluR2 and NR2B in the brain. This post-transcriptional inhibit the expression of GluR2 and NR2B genes by manipulating the sequence of GluR2 and NR2B gene 3'- UTR, further suppressing the expression of NMDA receptors, which could reduce NMDA mediated Ca^2+^ influx. Therefore, upregulation of miR-233 expression can inhibit the expression of NMDA, prevent excessive influx of Ca^2+^, thereby alleviating neuronal damage caused by intracellular Ca^2+^ overload, and further reduce neuronal cell damage in ischemic regions (Harraz et al., 2012). Following ischemic events, Endothelial cell-derived exosomal miRNAs play crucial roles in neuronal repair and regeneration (Huang et al., 2023). The brain has poor tolerance to ischemia and hypoxia, and after the blocking of a blood supply artery, irreversible necrosis and apoptosis of neuronal cells quickly occur. Emerging evidence demonstrates that exosomes derived from vascular endothelial cells played an important role in alleviating irreversible neuronal cell necrosis and apoptosis after ischemic events (Zhou Y. et al., 2022). In the study of cell models of ischemia-reperfusion injury in human umbilical vein endothelial cells (HUVECs) and human neuroblastoma cells (SH-SY5Y), exosomes derived from HUVECs are involved in regulating the proliferation, apoptosis, migration, and invasion of SH-SY5Y cells. Research found that exosomes containing CD63, HSP70, and tumor susceptibility gene 101 proteins are abundantly secreted into the cell supernatant and serum, while the expression of apoptosis related molecules caspase-3, Bax, and Bcl-2 in SH-SY5Y cells is reduced. The HUVECs-derived exosomes may inhibit SH-SY5Y cell apoptosis by regulating relevant signaling molecules in the apoptotic signaling pathway, and ultimately alleviate post ischemic injury (Teixeira et al., 2015). Research shows that the axons of neurons begin to regenerate around the infarcted area of acute ischemic stroke under stress stimulation. But they are also inhibited by the scar formed by astrocyte (Hira et al., 2018). Experimental evidence from a MCAO rat model reveals that astrocyte-secreted exosomes counteract axonal growth inhibition during oxygen-glucose deprivation. The results showed that the exosomes secreted by astrocyte promoted axonal regeneration by increasing the expression of prostaglandin D2 synthase (Hira et al., 2018).

### 4.2 Treatment of cerebral ischemic injury with exosomes

The contents of exosomes encapsulated by a lipid bilayer are protected from protease and nuclease degradation following cellular secretion. The exosomes, with their small structure and low immunogenicity can easily penetrate the BBB (Mittal et al., 2021), making exosomes particularly advantageous for treating neurological injury. Exosomes from different cells exhibit good therapeutic effects on brain injury (Li et al., 2018). Stem cell-derived exosomes facilitate neurovascular unit remodeling. The exosomes secreted by astrocytes, neural progenitor cells and microglia from the nervous system can promote nerve development, regeneration, and maintain neural stability. The exosomes from serum and plasma can prevent the destruction of the BBB (Gao et al., 2023), reduce reactive oxygen species (ROS), enhance superoxide dismutase (SOD) activity, and restore mitochondrial function, thereby reducing oxidative stress response and decreasing the area of cerebral infarction. Notably, the modification of the structure and content of exosomes by acupuncture also demonstrates good therapeutic potential (Gao et al., 2023). These findings position exosome-targeted drug development as a transformative strategy for brain injury treatment.

### 4.3 Exosomes improve cerebral ischemic injury through anti-inflammatory and anti apoptotic effects

MSCs have been widely utilized due to their multipotent differentiation potential. However, in MSC-based replacement therapy, only a small fraction of transplanted cells reach the injury site, and their post-implantation survival duration is limited, hindering the achievement of the desired therapeutic outcomes. To overcome these challenges, researchers have developed MSC-derived exosomes for the treatment of brain ischemic injury, which are better than stem cell replacement (Li et al., 2018; Gao et al., 2023). MSC-derived exosomes can improve brain ischemic injury by regulating brain cells and the microenvironment. Exosomes derived from human bone marrow MSCs were injected into the tail vein of brain ischemic injury model rats, resulting in improved learning and memory abilities. At the same time, the inflammatory factor IL-1α, IL-2 and tumor necrosis factor α (TNF- α) were significantly reduced in the serum, cortex, and hippocampus of the model rats. Additional research demonstrates that administering exosomes derived from rat bone marrow MSCs to brain ischemic injured rats, which reduced the cerebral infarction area of rats, promoted the remodeling of neurovascular units in rats, and induced M1 type microglia to polarize to M2 type, thereby improving the inflammatory response (Liu et al., 2024). Co-culture of hypoxia-preconditioned stem cells with microglia subjected to OGD/R enhances microglial survival while suppressing TNF- α, IL-1β and IL-6, and promote the expression of anti-inflammatory factor IL-10. Inhibiting the secretion of stem cell exosomes significantly alleviates the effect of stem cell culture on microglia (Yu et al., 2021). In MCAO/R model rat tail vein injection of exosomes derived from MSCs can reverse the M1 polarization of microglia mediated by cysteine leukotriene receptor two/extracellular signal regulated protein kinase, inhibit the inflammation of microglia, promote myelination and neuronal regeneration in rats, and significantly improve the motor, memory and learning abilities of rats (Zhao et al., 2020a). A large-scale case-control study demonstrated significantly elevated levels of exosomal miR-133 in acute stroke patients’ blood (Cuomo et al., 2024) within 72 h post-onset compared to controls, showing strong correlation with National Institutes of Health Stroke Scale score. MiR-133 can serve as a potential biomarker for ischemic brain injury (Chen et al., 2017). A study on miR-335 and calmodulin (CaM) in the exosomes of stroke patients found that the downregulation of miR-335 was closely associated with elevated plasma CaM levels, indicating a negative feedback regulatory mechanism between miR-355 and CaM signaling pathways (Mirzaei et al., 2018). At the same time, increasing evidence suggests that inhibiting the calcium calmodulin dependent protein kinase (CaMKK) signaling pathway reduces brain ischemic damage, suggesting that targeting the CaMKK pathway by upregulating miR-335 in the circulation could alleviate brain ischemic damage (McCullough et al., 2013). In a rat brain ischemic damage model, miR-122 was found in the exosomes of the peripheral circulation, which can improve the prognosis of rats (Li et al., 2018).

### 4.4 Exosome derived from nervous system cells improve cerebral ischemic injury through anti apoptosis

The nervous system comprises two principal cell types: neurons and glial cells. Exosomes derived from these nervous system cells carry diverse miRNAs (Table 3), which significantly influence brain ischemic injury pathophysiology. Recent studies have shown that exosomes secreted by healthy neural cells exert neuroprotective effects following brain ischemic injury. It has been found that astrocyte-derived exosomes downregulate mitogen-activated protein kinase/nuclear factor-κB by targeting Toll-like receptor 7, indicating the miR-34c’s critical role in mitigating ischemic neuronal damage (Wu et al., 2020). Exosomes derived from M2-polarized microglia are enriched with miR-137, which directly targets the Notch signaling pathway, reduces the apoptosis of OGD treated neurons *in vitro*, maintains neural homeostasis, and improves the infarct volume and behavior deficits of brain ischemic injured mice *in vivo* (Zhang D. et al., 2021). In MCAO model rats, intracerebroventricular administration of exosomes secreted by mouse brain microvascular endothelial cells significantly improved neurological function. After administration of exosomes, the migration and proliferation of neural progenitor cells increased, and apoptosis decreased (Zhou et al., 2020). Overexpression of miR-124 in M2 microglia-derived exosomes can alleviate ischemic brain damage and promote neuronal survival by inhibiting the expression of the downstream target protein specific protease (Song et al., 2019). In summary, exosomes derived from nervous system cells can improve neural communication, promote neuronal proliferation, inhibit apoptosis, and alleviate brain I/R injury.

**TABLE 3:** Exosomal microRNAs cargo in MSCs in ischemic stroke.

miRNA	Model	Proposed effects	References
miR-133b	MCAO	Neural remodeling	Zhang et al. (2021b)
miR-17–92	MCAO	Neural remodeling	Zhang et al. (2020)
miR-223-3p	MCAO	Anti-inflammation	Aldali et al. (2025)
miR-1906	MCAO	Anti-inflammation	Yang K. et al. (2022)
miR-132-3p	MCAO	BBB protection	Couch et al. (2017)
miR-21-3p	MCAO	BBB protection	Yang K. et al. (2022)
miR-184	MCAO	Neurogenesis	Yang K. et al. (2022)
miR-210	MCAO	Neurogenesis	Yang K. et al. (2022)
miR-126	MCAO	Neurogenesis	Xu et al. (2017)
miR-181b-5p	MCAO	Angiogenesis	Yang K. et al. (2022)
miR-124	MCAO	Neurogenesis	Song et al. (2019)
miR-22-3p	MCAO	Anti-apoptosis	Yang K. et al. (2022)
miR-146a-5p	MCAO	Anti-inflammation	Lamparski et al. (2002)

MCAO: middle cerebral artery occlusion; BBB: blood-brain barrier.

### 4.5 Exosomes from other sources improve cerebral ischemic injury through antioxidant stress and anti-inflammatory

Human blood also contains a large amount of exosomes, and research has confirmed that exosomes in the blood have anti-ischemic activity (Cuomo et al., 2024). After injection of plasma-derived exosomes into the brain of mice with ischemic injury, the generation of ROS in brain tissue decreases and the activity of SOD increases, which helps prevent the destruction of the BBB, inhibits cell apoptosis, and alleviate cerebral ischemic injury in mice (Jiang et al., 2020). When exosomes are extracted from mouse serum and co-cultured with neuroblastoma cells derived from an OGD/R model, they can effectively reduce inflammation, apoptosis, and oxidative stress, protecting neuroblastoma cells from OGD/R-induced damage. Overexpression of miR-451a in these exosomes further inhibits inflammation and oxidative stress (Li et al., 2021). Studies have found that exosomes derived from endothelial cells directly protect neurons from ischemic damage by promoting cell growth, migration, and inhibiting apoptosis (T et al., 2025). In summary, blood-derived exosomes can improve cerebral ischemic injury by reducing ROS production, increasing SOD activity, preventing BBB damage, and suppressing inflammation.

### 4.6 Exosomes improve cerebral ischemic injury through anti-ferroptosis

Ferroptosis is a novel type of cell death, characterized by iron-dependent excessive accumulation of lipid hydroperoxides. Ferroptosis has been proven to participate in many diseases, including cardiovascular disease (Wang W. et al., 2024), cancer (Yang et al., 2023), and neurological diseases (Ou et al., 2022). Acyl-CoA synthetase long-chain family member 4 (ACSL4) and glutathione (GSH), two regulators of ferroptosis, show different expression patterns during recovery from ischemic stroke (Tuo et al., 2022). Therefore, anti-ferroptosis therapy (e.g., Liproxstatin-1 and Ferrostatin-1) appears to be a promising treatment for ischemic stroke and neural repair. Exosomes containing biologically functional substances could repair neural cells when combined with intranasal administration. A study showed that adipose-derived exosomes can inhibit ferroptosis in neurons via the miR-760-3p/CHAC1 axis (Wang Y. et al., 2023), where miR-760-3p directly targets and downregulates CHAC1, a key enzyme promoting glutathione degradation, thereby preserving intracellular GSH levels and reducing lipid peroxidation (Wang Y. et al., 2023). In addition, Wang Y. et al. (2024) demonstrated that FXR2 in adipose-derived exosomes could regulate ATF3/SCL7A11 expression, alleviate ferroptosis in M2 microglia, and improve neurofunctional recovery after cerebral injury. FXR2 binds to ATF3 mRNA, stabilizing it and enhancing its transcription, which in turn upregulates SLC7A11, a critical component of the cystine/glutamate antiporter system (System Xc−), thus boosting glutathione synthesis and ferroptosis resistance. Qin et al. (Qin et al., 2024) reported that exosomal miR-Novel-3 derived from foam cells could induce neuroinflammation in microglia and macrophages by activating the TLR4/NF-κB pathway, exacerbating oxidative stress and indirectly promoting ferroptosis. Exosomes derived from umbilical cord-mesenchymal stem cells (UC-MSCs) containing circBBS2 enhanced Solute Carrier Family 7, Member 11 (SLC7A11) expression by sponging miR-494, thereby suppressing ferroptosis and alleviating ischemic stroke; circBBS2 acts as a competitive endogenous RNA (ceRNA) to sequester miR-494, which normally represses SLC7A11 translation, leading to increased System Xc− activity and glutathione production (Hong et al., 2023).

### 4.7 Application of modified exosomes in cerebral ischemic injury

The application of biological techniques allows for modification of the structure of exosomes, including their chemical ligands, proteins, lipids, and nucleic acids (Carnino et al., 2020), as well as modification of specific cargo substances in exosomes (Lu et al., 2021). Tissue engineering techniques can be used to quantify exosomes, offering a potential approach to effectively treat brain injury. Exosomes were isolated from rat whole blood, and quercetin and anti growth-associated protein-43 (GAP-43) monoclonal antibodies were integrated into the exosomes. Activation of the nuclear transcription factor erythroid 2-related factor 2 (Nrf2)/heme oxygenase-1 (HO-1) pathway significantly attenuates oxygen-glucose deprivation (OGD)-induced damage in human neuroblastoma cells. Intracerebral injection into MCAO/R model animals significantly enhances their brain delivery, promotes cell survival in the ischemic penumbra, and markedly inhibits ROS production (Guo et al., 2021). Additionally, exosomes derived from rat bone marrow MSCs that overexpress miR-223-3p can reduce infarct volume in MCAO/R model rats, improve neurological function, enhance learning and memory performance, and stimulate anti-inflammatory factor secretion in the ischemic cortex and hippocampus. *In vitro* studies demonstrate that miR-223-3p dose-dependently inhibits NMLTC4-induced M1 microglia polarization while promoting M2 microglia polarization (Zhao et al., 2020b). Several studies have isolated exosomes from bone marrow MSCs transfected with miR-26a-5p and administered them to MCAO model mice, resulting in a significantly reduced cerebral infarct volume. These exosomes can inhibit cyclin-dependent kinase six expression and markedly reduce apoptosis when co-cultured with OGD/R-induced mouse microglia (Cheng et al., 2021). An exosome linked to RAGE-binding peptide (RBP-Exo) was developed to generate engineered exosomes, Co-incubation of these engineered exosomes with neuroblastoma cells derived from hypoxia-treated mice significantly reduced the number of receptor for advanced glycation end products positive cells. When administered intranasally to brain injury model rats, these exosomes reduced pro-inflammatory cytokine TNF-α expression while also decreasing cell apoptosis and infarct volume (Kim et al., 2021). Multiple experimental results have demonstrated that exosomes could serve as potential biomarkers for brain injury. However, significant challenges remain in the purification and stable extraction of exosomes. Ultrafiltration technology (Table 4) has demonstrated significant potential for processing and analyzing blood-derived exosomes, though separation efficiency remains dependent on equipment specifications (Shirejini and Inci). Although the extraction and identification technology of exosome are still in the development stage, with the continuous progress of technology, the integration of sensors and micro units would be more multi-functional (Ahmed et al., 2020). Notably, plasma sensors can detect biological targets in real time and without labels, with unprecedented sensitivity and detection limits. These platforms can simultaneously isolate exosomes and perform on-chip analyses (Mataji-Kojouri et al., 2020). Emerging techniques for extracting, isolating, and identifying extracellular vesicles show significant promise for applications in brain injury treatment. MSCs have demonstrated therapeutic potential for stroke, with several MSC-based therapies currently undergoing clinical trials (Deng et al., 2019). Research has showed that MSCs can activate critical signaling pathways under cytokine stimulation, promoting nerve cell and vascular regeneration after stroke (Zhang et al., 2022). In addition, MSCs transport their functional proteins, mRNAs and miRNAs to distant recipient cells by secreting exosomes, which modulate cellular functions by enhancing or inhibiting the expression of related molecules in the target cells (Vahab et al., 2025). MSCs can also promote nerve growth by stimulating Schwann cells to secrete neurotrophic factors. Furthermore, studies have found that MSCs can differentiate into Schwann cells (Entezari et al., 2023). Endothelial-derived exosomes can effectively upregulate matrix metalloproteinase-1 (MMP-1), MMP-3 and nuclear factors κ B, promoting the proliferation, migration, and secretion activity of MSCs. Exosomes derived from MSCs play an active role in renal injury, liver injury, myocardial ischemia-reperfusion injury, and cerebral ischemic treatment (Zhou H. et al., 2022). The underlying mechanism may involve enhancing vascular integrity through endothelial cell repair, ultimately mitigating ischemia-reperfusion injury. Additionally, growing evidence suggests that exosomes from other stem cell sources also participate in cerebral ischemia pathogenesis (Zhang et al., 2023). As is well known, gene therapy can fundamentally treat diseases such as tumors and stroke. However, current limitations in plasmid construction accuracy, transfection, and *in vivo* targeting hinder its widespread application (Sudhakar and Richardson, 2019). Exosomes are considered excellent carriers for gene targeting delivery *in vivo*. After endocytosis by target cells, the genetic cargo in exosomes exerts a series of biological effects. Katakowski et al. (Katakowski et al., 2013) demonstrated that MSC-derived exosomes contain miR-146b, which can be internalized by glioma cells and subsequently inhibits their growth. Notably, even a small dose of miR-146b-enriched exosomes can inhibit the growth of glioma cells and alter their phenotype. In a rat cerebral ischemia model, injection of MSC-derived exosomes containing miR-133b into the ischemic brain area significantly improved neuronal plasticity (Aguiar Koga et al., 2023), indicating that bioactive molecules from MSC-derived exosomes entered neuronal cells and exerted functional effects. Further studies revealed that miR-133b could act on the target gene connective tissue growth factor (CTGF) and ras gene family A. During ischemic injury, miR-133b reduces the expression of CTGF in the ischemic region, thereby reducing the inflammatory response in the ischemic region (Xin et al., 2013).

**TABLE 4:** Current methods for isolation of exosoms

Method	Advantages	Disadvantage
Ultracentrifugation	1. Large-scale extraction 2. Simple operation 3. Widely used	1. Time-consuming 2. Costly instrumentation 3. Strict balancing before centrifugation
Ultrafiltration	1. High purity 2. Ultracentrifugation is not needed	1. Protein contaminants
Immunomagnetic bead	1. Rapid 2. Higher quantities 3. Greater sensitivity	1. Complex and difficult operation 2. Expensive
Commercial exosomes extract kit	1. Ultracentrifugation is not needed 2. Simple operation 3. Rapid 4. For rare sample	1. Expensive 2. Small-scale extraction

In fact, exosomes from MSCs, NSCs, and induced pluripotent stem cells (iPSCs) have been reported to be effective in tissue engineering repair, including skin regeneration, bone defect repair, and cerebral injury repair. Doeppner et al. (Doeppner et al., 2015) showed that exosomes originating from MSCs had significant effect on neurological recovery after stroke. The therapeutic function of exosomes is mainly dependent on the cargo in exosomes being transferred from MSCs to recipient cells, facilitating cell-to-cell communication. MiR-133b, a key small molecule in MSC-derived exosomes, has been proven to promote neurite growth (Aguiar Koga et al., 2023). In addition, miR-17–92 in MSC-derived exosomes could improve neurogenesis by targeting the PTEN/Akt pathway (Xin et al., 2017b). Similarly, miR-223-3p in MSC-derived exosomes promotes nerve repair through the CysLT2R-ERK1/2 pathway (Aldali et al., 2025). Meanwhile, miR-146a-5p in MSC-derived exosomes had a good effect on nerve repair by inhibition of the IRAK1/TNF pathway (Zhang Z. et al., 2021).

Recently, NSC-based therapy has been applied in regenerative treatment for cerebral injury. Similar to MSCs, NSC-derived exosomes could carry miR-206, miR-133a-3p, and miR-3656 and be taken up by target neuron cells, promoting nerve repair through the TGF-β1 and Bcl-2 pathways (Zhang et al., 2020).

## 5 Limitations

Although the application of miR-124-enriched MSC-derived exosomes is currently being evaluated in acute ischemic stroke patients (NCT03384433) (Aldali et al., 2025), several challenges remain for clinical application. First, the amount and types of exosomes vary in different cells and growth conditions, culture medium composition, and cell passage number, making batch-to-batch consistency difficult to achieve. Thus, a quality control standard for clinical-grade exosomes is needed, including defined criteria for purity, potency, surface markers, and cargo composition to meet regulatory requirements (Lamparski et al., 2002). Second, the advantages of exosomes in cerebral injury repair, such as lower immunogenicity, minimal oncogenicity, and the ability to cross the BBB, are widely established. However, the potential adverse effects of exosomes, including the dosage (optimal therapeutic concentration remains unclear), frequency (repeated dosing may trigger immune clearance), and administration routes (intranasal vs. intravenous delivery pharmacokinetics differ significantly) still lack consensus. Additionally, long-term safety concerns, such as unintended organ biodistribution and off-target effects of exosomal miRNAs, require further preclinical evaluation (Gurunathan et al., 2021). Third, current isolation methods, including ultracentrifugation (time-consuming and prone to protein contamination), size-based isolation (limited by exosome heterogeneity), immunoaffinity chromatography (expensive and antibody-dependent), and commercial reagent kits (low specificity), have their own advantages and disadvantages but cannot meet the requirements for large-scale production needed for clinical application due to low yield, high cost, and poor scalability. A standardized extraction method has not yet been established (Qu et al., 2023). Finally, although methods such as electroporation, sonication, freeze-thaw cycles, transfection, and extrusion have been used to improve the low delivery efficiency, the results are still insufficient for clinical application standards, as modified exosomes may exhibit reduced stability *in vivo* or unintended biological effects (Qu et al., 2023). Regulatory hurdles, including the lack of clear FDA/EMA guidelines for exosome-based therapies, further complicate clinical translation.

Future Directions such as emerging strategies—including CRISPR-engineered exosomes, biomimetic hybrid vesicles, and targeted modifications (e.g., RVG peptide for BBB penetration)—represent promising future directions to enhance delivery precision and therapeutic efficacy. Additionally, longitudinal studies tracking exosome biodistribution and immune responses in non-human primates will be critical to de-risk clinical translation. In addition, many miRNA (Yang K. et al., 2022) involved in ischemic brain injury were not listing in this review.

## 6 Conclusion

MSCs play a significant therapeutic role in ischemic stroke by promoting neural function recovery following cerebral ischemia injury. Notably, exosomes derived from MSCs contain multiple active components that demonstrate significant neuroprotective effects in brain injury. These nanovesicles exhibit low immunogenicity, minimal toxicity, and efficient targeted cell uptake. Through structural modification, MSC-derived exosomes can enhance drug delivery to the lesion site, thereby significantly improving therapeutic efficacy for brain injuries. Furthermore, exosomes and their cargo of bioactive substances function as intercellular messengers, facilitating communication between neuronal cells and vascular endothelial cells during post-stroke damage and repair processes Current preparations for clinical trials are underway and showing promising preliminary results (Deng et al., 2019). However, critical questions remain regarding how these modifications affect exosome stability, cellular internalization pathways, and *in vivo* tissue distribution, which require further elucidation.

## Abbreviations

miRNA, MicroRNA; MSCs, Mesenchymal stem cells; NVU, Neurovascular unit; ECM, extracellular matrix; BBB, blood brain barrier; VEGF, Vascular endothelial growth factor; bFGF, basic fibroblast growth factor; TGF- β: transforming growth factor- β; NSCs, neural stem cells; NPCs, neural progenitor cells; SVZs, subventricular zone; SGZs, subgranular zone; IGF-1, insulin-like growth factor-1; BDNF, brain-derived neurotrophin; ANG 1: angiopoietin 1 MCAO, middle cerebral artery occlusion; PI3K, Phosphatidylinositol-3-kinase; SDF-1 α: Stromal cell derived factor-1 α; CXCR4, chemokine receptor 4; IL-1, interleukin-1; OGD/R, oxygen deprivation/reperfusion; TLR, Toll like receptor; ACSL4, acyl-CoA synthetase long-chain family member 4; GSH, glutathione; Growth-associated protein-43 (GAP43); SLC7A11, Solute Carrier Family 7, Member 11.
